# Cutaneous vesicles in an infant after bone marrow transplantation

**DOI:** 10.1002/jha2.504

**Published:** 2022-07-27

**Authors:** Maria A. Pereda, Kelly Scarberry, Jignesh Dalal

**Affiliations:** ^1^ Pediatric Hematology Oncology and Bone Marrow Transplantation Case Western Reserve University Cleveland Ohio USA; ^2^ Rainbow Babies & Children's Hospital Cleveland Ohio USA; ^3^ University Hospitals Cleveland Ohio USA

1

Eight‐month‐old infant who recently received a matched unrelated donor transplant for malignant infantile osteopetrosis was admitted on T+48 for the evaluation of fevers. Her complete blood count (CBC) on admission showed a neutrophil count of 8940 × 10^9^/L and Hb of 7.1 g/dl. Patient was started on cefepime and vancomycin while awaiting results of blood culture that grew methicillin‐susceptible *Staphylococcus aureus*. She continued treatment with cefepime and had her central line removed. On T+50 parents reported the appearance of a new blistering rash on her hands. Skin examination demonstrated multiple 1–2 mm deep seated noninflammatory vesicles located on the distal digits and metacarpophalangeal joints of both hands and pad of the right thumb (Figure 1). During this admission, our patient's Epstein–Barr virus (EBV) polymerase chain reaction (PCR) test in whole blood became positive and she also had diarrhea that tested positive for *norovirus*.

**FIGURE 1:**
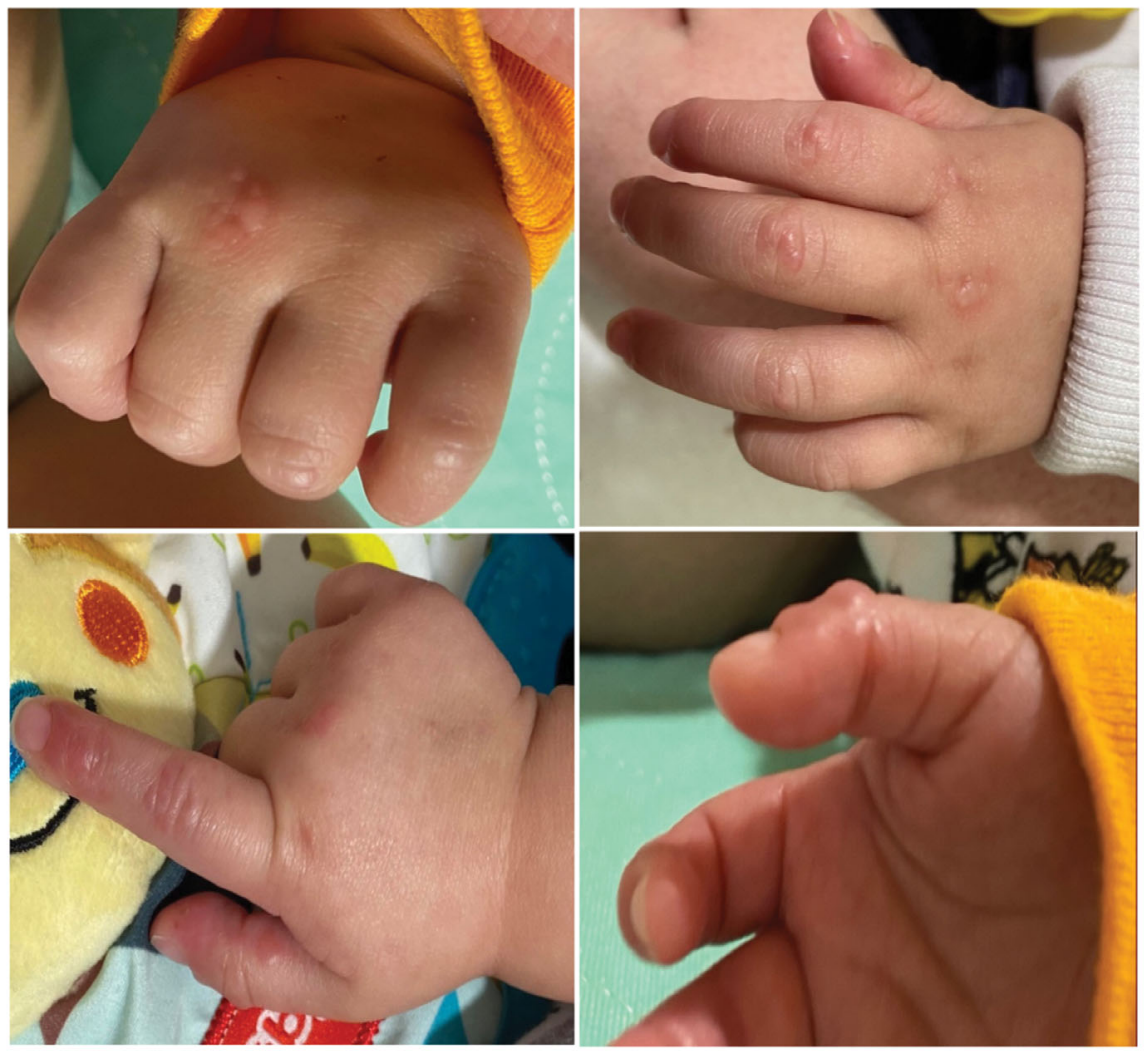
Bilateral hand involvement with multiple 1–2 mm noninflammatory vesicles mostly located over metacarpophalangeal joints

In an early posttransplant (<100 days) patient, our major concern was acute graft versus host disease (GVHD). Due to the limited distribution of rash and the lack of systemic symptoms, it became less likely. Although GVHD can present as bullous skin lesions, it is associated with severe disease presentation. Second in our list were infectious causes. There are reports of *norovirus*‐related urticaria presenting with generalized erythematous wheals associated with pruritus. Dermatologic manifestations of EBV are reported more commonly in patients with infectious mononucleosis (IM). IM patients more commonly develop a faint erythematous macular rash early in the course or a maculopapular rash associated with the inappropriate use of amoxicillin. There are reports of IM presenting with erythematous papulovesicules, but it is uncommon. Vesicular lesions associated with Herpes simplex 1 or 2 infection are characterized by grouped vesicles on an erythematous base ring. In severely immunocompromised patients with deficient cellular immunity, the risk of extensive mucocutaneous disease and disseminated infection is higher. Transplant patients are at risk of developing *herpes simplex* virus (HSV) infection due to reactivation. Lastly, sucking blisters are noninflammatory vesicles with thick walls that contain sterile fluid. Lesions are usually located on dorsal areas of fingers, hands or wrists and can be bilateral. The observation of the infant sucking the involve areas helped guiding with the diagnosis. In the absence of lesions in other areas and with lack of systemic symptoms sucking blisters became the most likely diagnosis. No biopsy was performed due to well apearing baby. Fever subsided and antibiotics were stopped after 10 days with negative blood culture. Rash is persistent with a healthy looking infant without other manifestations.

## CONFLICT OF INTEREST

The authors declare they have no conflicts of interest.

## FUNDING INFORMATION

The authors received no specific funding for this work.

## ETHICS STATEMENT

A written informed consent was obtained from the parents of the patient to publish the case and images in a medical journal.

